# The status of *Bactrocera invadens* Drew, Tsuruta & White (Diptera: tephritidae) inferred from complete mitochondrial genome analysis

**DOI:** 10.1080/23802359.2016.1219638

**Published:** 2016-09-03

**Authors:** Li-Jie Zhang, Li-Hui Jiang, Chun-Yan Wei, Ruo-Si Liu, Xing-Liang Liu, Jian-Guang Li, Huai-Jun Xue

**Affiliations:** a Beijing Entry-Exit Inspection and Quarantine Bureau, Beijing, China;; b Jilin Entry-Exit Inspection and Quarantine Bureau, Changchun, China;; c Key Laboratory of Zoological Systematics and Evolution, Institute of Zoology, Chinese Academy of Sciences, Beijing, China

**Keywords:** Mitochondrial genome, phylogeny, *Bactrocera invadens*, *Bactrocera dorsalis*

## Abstract

In this study, the complete 15,911 bp mitochondrial genome (mitogenome) of *Bactrocera invadens* was sequenced. To estimate the status of *B. invadens*, all available mitogenomes of *Bactrocera* were downloaded from GenBank for phylogenetic analysis. Phylogenetic analyses showed that *B. invadens*, *B. philippinensis*, *B. papayae*, and three *B. dorsalis* sequences formed a well-supported clade with very short terminal branch lengths, indicting the relatively close evolutionary relationships of these taxa. The results further supported that *B. invadens*, the same as *B. philippinensis* and *B. papayae*, belongs to the same species as *B. dorsalis*.

*Bactrocera invadens* Drew, Tsuruta & White belongs to Dacinae of Tephritidae, a subfamily which includes a large number of widely distributed and damaging horticultural pest species. This species was first detected in Africa in 2003 and has since become a destructive and highly invasive species, attacking over 40 fruit species and recorded from more than 30 African countries (Lux et al. 2003; Drew et al. 2005; Goergen et al. 2011; Khamis et al. 2012; Schutze et al. 2015). *Bactrocera invadens* is widely regarded as one of *B. dorsalis* complex. It is morphologically very similar to *B. dorsalis*. Furthermore, *B. dorsalis* and *B. invadens* shared the same COI haplotype (Schutze et al. 2015) and the close original place. *Bactrocera invadens* originates from the Indian subcontinent and has recently invaded all of sub-Saharan Africa, and *B. dorsalis* principally occurs from the Indian subcontinent towards southern China and South-east Asia. High morphological and genetic similarity has cast doubt over whether *B. invadens* is a distinct species from *B. dorsalis* (Schutze et al. 2015).

The specimens used in this study were intercepted from mangoes from Kenya and deposited in the plant laboratory of Beijing Entry-Exit Inspection and Quarantine Bureau. The complete mitochondrial genome (mitogenome) of *B. invadens* is a double-stranded circular molecule of 15,911 bp in length (GenBank accession number KX534207), with 22 transfer RNA genes, 13 protein-coding genes, 2 ribosomal RNA genes, and a control region as in other insects. The overall base composition is A: 39.30%, T: 34.33%, C: 16.21%, and G: 10.16%, with a much higher A + T content. To estimate the status of *B. invadens*, all available mitogenomes of *Bactrocera* (accession numbers: DQ845759, DQ917577, KM244662, DQ995281, DQ917578, EF014414, HQ130030, KP296150, JX456552, KR233259, GU108478) were selected as ingroups and *Ceratitis capitata* (accession number: AJ242872) was selected as an outgroup. The phylogenetic tree was reconstructed using the Mrbayes v3.2.2 (Mrbayes Inc., La Jolla, CA) (Ronquist et al. 2012) based on the nucleotide sequences of the 13 protein-coding genes. The aligned data from each locus were concatenated with Sequence Matrix v.1.7.8 (Vaidya et al. 2011). The best-fit models of nucleotide substitution for the 13 protein-coding genes were selected using the Akaike Information Criterion (AIC) in jModelTest 0.1.1 (Posada 2008). Four independent Markov chains were run for 3 million Metropolis-coupled generations, with tree sampling occurring every 100 generations and a burn-in of 25% trees and posterior probabilities were estimated for each node.

Phylogenetic analyses showed that *B. invadens*, *B. philippinensis*, *B. papayae*, and three *B. dorsalis* sequences formed a well-supported clade with very short terminal branch lengths (Figure 1), indicating the relatively close evolutionary relationships of these taxa. Some individuals of *B. dorsalis* possessed haplotypes more closely related to *B. invadens* than to conspecifics. The results further supported that *B. invadens*, the same as *B. philippinensis* and *B. papayae*, belongs to the same species as *B. dorsalis*, which was proposed by some previous studies (e.g. Khamis et al. 2012; Frey et al. 2013; Leblanc et al. 2013; Jose et al. 2013; Schutze et al. 2015).

**Figure 1.**
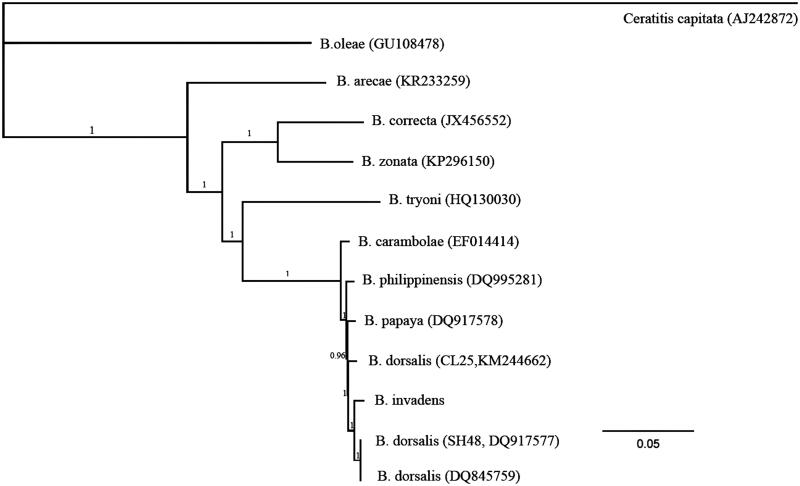
The Bayesian phylogenetic tree of genus *Bactrocera* (Tephritidae: Dacinae) based on the mitochondrial genome sequences. The values above the branches represent the Bayesian posterior probabilities.
